# Real-World Clinical and Economic Outcomes Among Patients with Localized Prostate Cancer Treated with External Beam Radiation Therapy Across Different Risk Stratifications in the United States

**DOI:** 10.36469/001c.171930

**Published:** 2026-10-30

**Authors:** Neal Shore, Benjamin Lowentritt, Charmi Patel, Sabree Burbage, Frederic Kinkead, Carmine Rossi, Francesca Lee, Dominic Pilon, Lawrence Karsh, Gordon Brown

**Affiliations:** 1 START Carolinas/Carolina Urologic Research Center, Myrtle Beach, South Carolina, USA; 2 Chesapeake Urology, Towson, Maryland, USA; 3 Johnson & Johnson, Horsham, Pennsylvania, USA; 4 Analysis Group, Inc., Montréal, Canada; 5 Advent Health Urology, Denver, Colorado, USA; 6 New Jersey Urology, Cherry Hill, New Jersey, USA

**Keywords:** external beam radiation therapy, healthcare costs, localized prostate cancer, risk stratification, survival

## Abstract

**Background:**

External beam radiation therapy (EBRT) is an effective treatment option for patients with localized prostate cancer (LPC). Despite the widespread use of EBRT, evaluation of outcomes based on LPC risk status is limited.

**Objectives:**

To comprehensively compare clinical and economic outcomes between patients with high-risk vs low/intermediate-risk LPC following EBRT in real-world United States (US) clinical practice.

**Methods:**

This retrospective study used linked patient-level electronic medical record data from PPS Analytics and administrative claims data from the Komodo Research Database (1 January 2016–31 August 2024). Patients with LPC treated with EBRT (date of the first claim was defined as the index date) were identified and stratified into high-risk and low/intermediate-risk cohorts based on clinical guidelines incorporating information based on localized cancer staging, Gleason score, and prostate-specific antigen level. Baseline characteristics were balanced using inverse probability of treatment weighting. A weighted Cox proportional hazards model was used to compare metastasis-free survival (MFS) and event-free survival (EFS) between cohorts. Weighted ordinary least squares regression was used to compare all-cause and PC-related healthcare costs (2024 US dollars), measured per-patient-per-year (PPPY), in the subgroup of patients with continuous insurance eligibility.

**Results:**

In total, 5984 and 10 471 patients were included in the high-risk and low/intermediate-risk LPC cohorts, respectively. Patients with low/intermediate-risk vs high-risk LPC had significantly longer MFS (36-month hazard ratio [HR]; 95% confidence interval [CI]: 2.47 [2.15, 2.84], *P* < .001; 60-month HR: 2.29 [2.05, 2.55], *P* < .001) and EFS (36-month HR: 1.84 [1.65, 2.06], *P* < .001; 60-month HR: 1.90 [1.73, 2.08], *P* < .001). Among the subgroup of patients with continuous insurance eligibility (high-risk: 980 patients; low/intermediate-risk: 1862 patients), patients with high-risk LPC incurred significantly higher mean total all-cause healthcare costs (cost difference [95% CI]: $8269 PPPY [3869, 12 800], *P* < .001) and mean total PC-related healthcare costs ($9053 PPPY [5232, 13 147], *P* < .001) post-index compared with those with low/intermediate-risk LPC.

**Conclusion:**

Patients with high-risk vs low/intermediate-risk LPC had significantly worse survival outcomes and incurred significantly higher costs following EBRT, consistent with the greater disease burden associated with high-risk disease. More effective treatment strategies are needed to further improve clinical outcomes and reduce economic burden among this patient population.

## INTRODUCTION

An estimated 1 in 8 men in the United States (US) will be diagnosed with prostate cancer (PC) during their lifetime, and over 300 000 cases were expected to be newly diagnosed in 2025.^1,2^ Advances in PC detection, including the widespread use of prostate-specific antigen (PSA) testing and the integration of multiparametric magnetic resonance imaging into diagnostic pathways, have increased early diagnosis, and nearly 70% of cases are now identified at the localized stage.^3,4^

Localized PC (LPC) refers to disease confined to the prostate that has not spread to regional lymph nodes or distant sites, and is stratified into low-, intermediate-, high-, or very high–risk categories based on tumor node metastasis (TNM) staging, PSA levels, and Gleason score, per the NCCN Clinical Practice Guidelines in Oncology (NCCN Guidelines®).^5^ Management is guided primarily by risk group but should additionally incorporate shared decision-making that considers comorbidities, life expectancy, and patient preferences.^6,7^ Both the NCCN Guidelines and the American Urological Association (AUA) recommend active surveillance as the preferred approach for most patients with low-risk LPC.^5,7^ Radical prostatectomy or radiation therapy (RT) may be appropriate for select low-risk LPC cases including those with high-volume cancer or higher PSA density.^5,7^ For patients with intermediate- or high-risk LPC, treatment typically involves radical prostatectomy or RT, with or without brachytherapy or androgen-deprivation therapy (ADT).^5,7^ RT most often consists of external beam RT (EBRT), delivered using techniques such as 3-dimensional conformal RT, intensity modulated RT, or stereotactic body RT.^8^ As both radical prostatectomy and EBRT are well-established and effective treatment options, selection is often driven by patient preference regarding treatment logistics and potential toxicity.^9^

Despite the widespread use of EBRT for LPC, there is limited existing literature describing clinical and economic outcomes stratified by risk group. Among the few studies that have examined this topic, high-risk vs low/intermediate-risk LPC was associated with a higher risk of advanced PC therapy use, metastasis, and death,^10,11^ as well as higher medical costs.^12^ Accordingly, the present real-world study aimed to comprehensively evaluate clinical and economic outcomes among patients with high-risk vs low/intermediate-risk LPC treated with EBRT in US clinical practice.

## METHODS

### Data Source

Linked patient-level data spanning January 1, 2016, to August 31, 2024, were obtained from 2 unique data sources: a clinical electronic medical record (EMR) database (Precision Point Specialty Analytics; PPS) and an administrative claims database (Komodo Research Database; KRD+).

PPS contains a wide range of demographic and clinical data collected as part of routine urology practice in the US, including PSA results, dispensation information, and PC-specific medication and procedure data not available from traditional administrative databases. In contrast, KRD+ is an insurance claims database comprising open and closed claims for >320 million US patients across commercial, Medicaid, and Medicare insurers. It includes information on insurance eligibility, diagnoses and procedures received in inpatient and outpatient settings, and prescription fills. Notably, mortality data captured in KRD+ are obtained from multiple third-party sources that aggregate information from national and state governments, public listings, private claims, and obituaries and are therefore not dependent on data from healthcare providers.

Linkage of patient-level PPS and KRD+ data was performed by Datavant using their patent-pending, machine learning–validated, de-identification technology in which private patient information is replaced with an encrypted token that cannot be reverse-engineered to reveal the original information.

This study was considered exempt research under 45 CFR § 46.104(d)(4) as it involved only the secondary use of data that were de-identified in compliance with the Health Insurance Portability and Accountability Act (HIPAA), specifically, 45 CFR § 164.514.

### Study Design

This study used a retrospective, longitudinal design to analyze clinical and cost outcomes among high-risk and low/intermediate-risk cohorts of patients with LPC who received EBRT (**Supplementary Figure S1**).

Adult male patients were required to have initiated EBRT on or after July 1, 2016, with the index date defined as the date of the first record for EBRT. To restrict the study population to patients with LPC treated with EBRT, patients were excluded based on having any of the following clinical indicators prior to or on the index date: (1) evidence of metastasis, to restrict the study population to patients with localized disease; (2) diagnosis of non-prostate cancer, to restrict the study population to patients with PC; (3) use of other localized therapy for LPC (ie, cryotherapy, interstitial prostate brachytherapy, high-intensity focused ultrasound), to restrict the study population to patients treated with EBRT; (4) use of advanced PC-related therapy (ie, androgen receptor pathway inhibitors [ARPIs], chemotherapy, immunotherapy, estrogens, radiopharmaceuticals, poly [ADP-ribose] polymerase [PARP] inhibitors), as these therapies are indicated for advanced or metastatic disease; or (5) evidence of castration resistance, as this reflects progression beyond localized, hormone-sensitive disease. Patients were also required to have ≥12 months of insurance claim activity in KRD+ or clinical activity in PPS pre-index. In the overall population meeting all criteria above, a subgroup of patients with ≥12 months of continuous insurance eligibility in KRD+ prior to and including the index date were identified for the assessment of economic outcomes.

Patients were classified into mutually exclusive high-risk and low/intermediate-risk cohorts based on their LPC risk level (**Supplementary Table S1**). Risk was determined according to TNM staging, PSA level, and Gleason score during the 180 days pre-index, in alignment with the NCCN Guidelines.^5^ However, not all patients had records for all 3 clinical variables, and thus risk classification was performed using the minimum available data (eg, patients with a PSA level of >20 ng/mL were classified as high-risk regardless of whether other tests were available). The proportion of patients with an LPC risk assessment determined by 1, 2, or all 3 clinical variables is reported in **Supplementary Table S2**.

The baseline period included 12 months prior to the index date. Clinical outcomes were assessed during an observation period spanning from the index date until the latter of end of claim activity in KRD+ or clinical activity in PPS (including death), both no later than the end of data availability (ie, August 31, 2024). Cost outcomes were assessed during an observation period spanning from the index date until the earliest of end of continuous insurance eligibility or end of data availability. The LPC period was defined as the time from the index date until the earliest evidence of disease progression, defined as metastasis, castration-resistance, or biochemical recurrence (BCR). BCR was defined as an increase of ≥2 ng/mL from the nadir PSA value observed after treatment and was evaluated from the index date until the earliest of metastasis, initiation of advanced PC-related therapy (ie, ARPIs, chemotherapy, immunotherapy, radiopharmaceuticals, PARP inhibitors, estrogen), castration resistance, or end of clinical activity in PPS.^13^ The date of the increase defined the BCR date. The nadir PSA value was time-updated, meaning it changed in time to the lowest PSA measurement captured during the observation period. The index EBRT-treatment period was defined as the first 90 days of the observation period starting on the index date (inclusively).

### Study Outcomes

Demographic (ie, age, race, geographic region, payer type) and clinical characteristics (ie, time between PC diagnosis and index date, ADT use overlapping the index date, prior first-generation ARPI use, prior bone antiresorptive therapy, Quan-Charlson Comorbidity Index [Quan-CCI], comorbidities, digital rectal examination, most recent PSA result, most recent TNM staging information, testosterone testing, earliest Gleason score, conventional and next-generation imaging tests) were evaluated prior to or on the index date.

Metastasis-free survival (MFS) and event-free survival (EFS) were evaluated among the overall patient population. MFS was defined as the time from the index date to the earliest date of metastasis or death from any cause, while EFS was defined as the time from the index date to the earliest date of BCR, metastasis, or death from any cause.

Total all-cause and PC-related healthcare costs were evaluated among the subgroup of patients with continuous insurance eligibility and reported per-patient-per-year (PPPY). PC-related costs were identified based on *International Classification of Diseases, 10th Revision, Clinical Modification* (ICD-10-CM) code C61 and procedure codes for luteinizing hormone-releasing hormone or other therapies for PC recommended by the NCCN Guidelines.^5^

Costs were reported in 2024 US dollars (USD) by adjusting for inflation using the medical care component of the US Consumer Price Index, and reported as imputed allowed amounts, reflecting the total payer and patient costs from the KRD+. Components of total healthcare costs were reported based on service type and included pharmacy and medical (ie, inpatient, outpatient, emergency room, and other services) costs. All cost variables were truncated at the 99th percentile to account for the influence of extreme outliers.

Costs were reported during the baseline, observation, and LPC periods, separately. Additionally, index treatment costs, including index procedure costs (ie, claims with a procedure code for EBRT) and index procedure-related costs (ie, claims with a procedure code for medical acts directly related to EBRT), were reported during the index treatment period.

### Statistical Analysis

Baseline variables were balanced between the high-risk and low/intermediate-risk cohorts using inverse probability of treatment weighting (IPTW). Propensity scores (PS) were generated using probability estimates from a logistic regression model based on dependent (ie, high-risk) and independent (ie, age, race, geographic region, payer type, year of index date, time between PC diagnosis and index date, use of first-generation ARPI, use of bone antiresorptive therapy, and baseline Quan-CCI) variables. Patients in the high-risk and low/intermediate-risk cohorts were attributed a weight of 1/PS and 1/(1-PS), respectively, with weights normalized using the mean weight of each respective cohort and truncated to the 95th percentile.^14^ No patients were removed from the study due to use of IPTW. The weighted cohorts were considered well-balanced if individual baseline characteristics had a standardized difference of <10%.^15^ Two separate PS models and set of weights were generated for the overall population (ie, comparison of clinical outcomes) and the subgroup of patients with continuous insurance eligibility (ie, comparison of cost outcomes).

Continuous variables were reported using means, standard deviations (SDs), and medians, and categorical variables were reported using frequencies and proportions. Among the clinical outcome population, weighted Kaplan-Meier (KM) analyses were used to assess MFS and EFS, separately, for the high-risk and low/intermediate-risk cohorts. A weighted Cox proportional hazards model was used to calculate hazard ratios (HRs) and 95% confidence intervals (CIs) comparing MFS and EFS between the high-risk and low/intermediate-risk cohorts. Results were reported at 36- and 60-months post-index.

Among the subgroup of patients with continuous insurance eligibility, total all-cause and PC-related healthcare costs during the observation period were compared between the high-risk and low/intermediate-risk cohorts using mean cost differences, estimated based on weighted ordinary least squares regression. To account for skewness in the distribution of cost outcomes, the 95% CIs and *P* values for mean cost differences were estimated using nonparametric bootstrapping procedures with 500 replications.

SAS Enterprise Guide software Version 7.15 (SAS Institute) was used to perform all analyses.

## RESULTS

### Patient Characteristics

**Overall population:** Among patients with LPC treated with EBRT, 5984 were included in the high-risk cohort and 10 471 were included in the low/intermediate-risk cohort (**Figure 1**). Baseline characteristics were largely well-balanced after weighting (ie, standardized difference <10%). The mean (median) age was 70.8 (71.0) years in the high-risk cohort and 70.5 (71.0) years in the low/intermediate-risk cohort. Among the high-risk and low/intermediate-risk cohorts, 49.5% and 49.7% of patients, respectively, were White and 17.1% and 16.8% of patients, respectively, were Black or African American (**Table 1; Supplementary Table S3**). Most patients in each cohort were insured with Medicare (high-risk: 76.7%; low/intermediate-risk: 76.0%). The mean (median) Quan-CCI was similar in both cohorts (high-risk: 3.3 [3.0]; low/intermediate-risk: 3.2 [3.0]).

**Figure 1.**
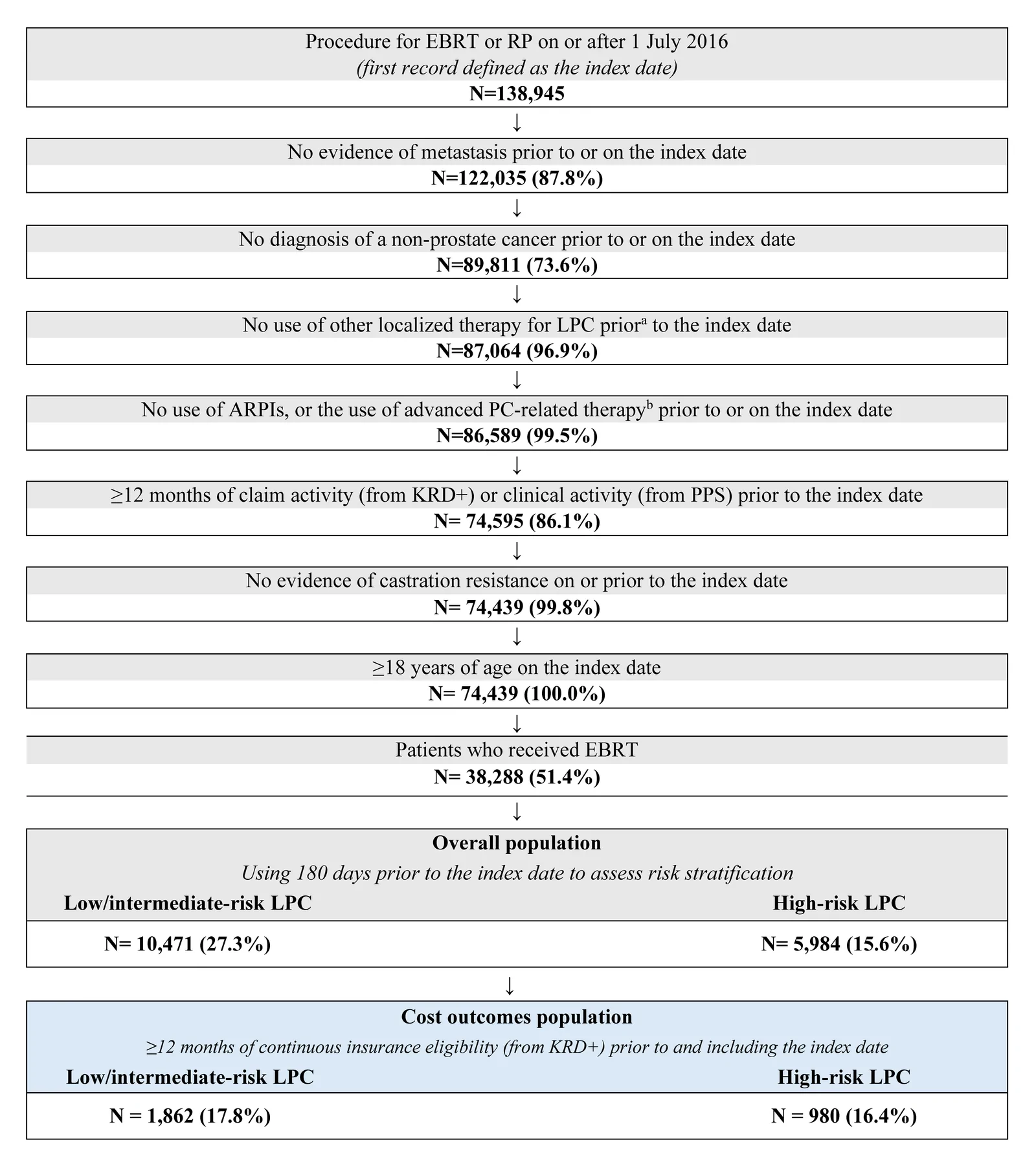
Patient Selection Flowchart Abbreviations: ARPI, androgen receptor pathway inhibitor; EBRT, external beam radiation therapy; KRD+, Komodo Research Database; LPC, localized prostate cancer; PARP, poly ADP-ribose polymerase; PC, prostate cancer; PPS, Precision Point Specialty; RP, radical prostatectomy. ^a^Other therapies for LPC included cryotherapy, interstitial prostate brachytherapy, and high-intensity focused ultrasound. ^b^Advanced PC-related therapies included ARPIs, chemotherapy, immunotherapy, estrogens, radiopharmaceuticals, and PARP inhibitors.

**Table 1.** Baseline Characteristics of Patients with LPC Treated with EBRT

	**Weighted Population^a,b^**					
**Overall**			**Subset of Patients with Closed Claims Eligibility**			
**High-Risk (N = 5984)**	**Low /Intermediate-Risk (N = 10 471)**	**Std Diff,^c^ %**	**High-Risk (N = 980)**	**Low/Intermediate-Risk (N = 1862)**	**Std Diff,^c^ %**	
Age, mean ± SD [median]	70.8 ± 7.3 [71.0]	70.5 ± 7.2 [71.0]	4.4	68.9 ± 8.0 [69.0]	68.6 ± 7.6 [69.0]	5.0
Race/ethnicity, n (%)						
White	2962 (49.5)	5205 (49.7)	0.4	442 (45.1)	852 (45.8)	1.3
Black or African American	1021 (17.1)	1761 (16.8)	0.7	188 (19.2)	339 (18.2)	2.5
Asian or Pacific Islander	98 (1.6)	154 (1.5)	1.4	14 (1.4)	25 (1.4)	0.5
Hispanic or Latino	47 (0.8)	85 (0.8)	0.3	9 (1.0)	19 (1.0)	0.6
Unknown	1856 (31.0)	3267 (31.2)	0.4	326 (33.3)	626 (33.6)	0.7
Geographic region, n (%)						
South	3255 (54.4)	5688 (54.3)	0.1	466 (47.6)	880 (47.2)	0.7
Midwest	1292 (21.6)	2297 (21.9)	0.9	240 (24.5)	464 (24.9)	0.9
Northeast	1000 (16.7)	1746 (16.7)	0.1	194 (19.8)	367 (19.7)	0.3
West	436 (7.3)	737 (7.0)	1.0	79 (8.1)	150 (8.1)	0.0
Payer type, n (%)						
Medicare	4592 (76.7)	7957 (76.0)	1.7	605 (61.8)	1126 (60.5)	2.7
Commercial	1083 (18.1)	1979 (18.9)	2.1	299 (30.5)	595 (31.9)	3.2
Medicaid	110 (1.8)	186 (1.8)	0.4	39 (4.0)	75 (4.0)	0.2
Unknown	200 (3.3)	349 (3.3)	0.1	37 (3.8)	66 (3.6)	1.1
Year of EBRT procedure (index year), n (%)						
2017	763 (12.7)	1341 (12.8)	0.2	97 (9.9)	184 (9.9)	0.2
2018	840 (14.0)	1501 (14.3)	0.9	145 (14.8)	285 (15.3)	1.3
2019	907 (15.2)	1568 (15.0)	0.5	148 (15.1)	273 (14.6)	1.2
2020	818 (13.7)	1417 (13.5)	0.4	140 (14.3)	257 (13.8)	1.5
2021	1007 (16.8)	1739 (16.6)	0.6	164 (16.7)	312 (16.7)	0.0
2022	913 (15.2)	1584 (15.1)	0.4	174 (17.8)	326 (17.5)	0.8
2023	720 (12.0)	1284 (12.3)	0.7	109 (11.1)	224 (12.1)	3.1
2024	16 (0.3)	33 (0.3)	0.9	2 (0.2)	2 (0.1)	2.6
Time between initial PC diagnosis and index date, mo, mean ± SD [median]	11.6 ± 23.2 [3.9]	12.4 ± 23.5 [3.6]	3.5	11.5 ± 21.4 [3.9]	12.6 ± 24.4 [3.6]	4.6
Prior use of ADT, n (%)	4859 (81.2)	4668 (44.6)	81.9*	791 (80.8)	786 (42.2)	86.3*
Use of first-generation ARPI, n (%)	549 (9.2)	823 (7.9)	4.7	109 (11.1)	172 (9.2)	6.3
Use of bone antiresorptive therapy, n (%)	194 (3.2)	264 (2.5)	4.3	23 (2.4)	36 (1.9)	3.0
Quan-Charlson Comorbidity Index, mean ± SD [median]	3.3 ± 1.7 [3.0]	3.2 ± 1.7 [3.0]	1.8	3.6 ± 1.9 [3.0]	3.5 ± 1.9 [3.0]	2.4

Abbreviations: ADT, androgen deprivation therapy; ARPI, androgen receptor pathway inhibitor; EBRT, external beam radiation therapy; LPC, localized prostate cancer; PC, prostate cancer; SD, standard deviation; Std Diff, standardized difference.

^a^The propensity score was obtained from a logistic regression model where index treatment was the dependent variable and with the following baseline characteristics as independent variables: age, race, geographic region, payer type, year of index date, time between PC diagnosis and index date, use of first-generation ARPI, use of bone antiresorptive therapy, and Quan-Charlson Comorbidity Index score.

^b^The number of patients reported in this weighted population represents the sum of weights for the corresponding nonweighted patients, rounded to the nearest integer. The proportions displayed were calculated before the rounding and may be slightly different than if they were calculated based on rounded numbers.

^c^A standard difference of <10% was considered well balanced.

*Std Diff ≥10%.

### Cost Outcomes Population

A subset of 980 patients in the high-risk cohort and 1862 patients in the low/intermediate-risk cohort were selected for the evaluation of cost outcomes. After weighting, baseline characteristics were largely well-balanced between cohorts (ie, standardized difference <10%). The mean (median) age was 68.9 (69.0) years and 68.6 (69.0) years in the high-risk and low/intermediate-risk cohorts, respectively. In the high-risk cohort, 45.1% of patients were White and 19.2% of patients were Black or African American, while in the low/intermediate-risk cohort, 45.8% of patients were White and 18.2% of patients were Black or African American. The mean (median) Quan-CCI was 3.6 (3.0) and 3.5 (3.0) in the high-risk and low/intermediate-risk cohorts, respectively. The mean (median) number of days patients received EBRT during the observation period was 37.6 (41.0) days in the high-risk cohort and 35.3 (40.0) days in the low/intermediate-risk cohort.

The mean age of patients in the cost outcomes population was numerically similar to that in the overall population, as were the proportions of patients who were White and Black or African American, respectively. While Medicare was the most common payer type in both the overall and cost outcomes populations, a numerically higher proportion of patients in the cost outcomes population were insured commercially.

During the baseline period mean (median) total all-cause healthcare costs were $14 557 ($11 416) PPPY in the high-risk cohort and $13 017 ($10 030) PPPY in the low/intermediate-risk cohort, including mean (median) all-cause pharmacy costs of $693 ($41) PPPY and $757 ($41) PPPY, respectively, and mean (median) all-cause medical costs of $13 801 ($10 819) PPPY and $12 100 ($9319) PPPY, respectively (**Supplementary Table S4**). Mean (median) total PC-related healthcare costs were $8579 ($6818) PPPY in the high-risk cohort and $7268 ($5666) PPPY in the low/intermediate-risk cohort.

### Clinical Outcomes

Within the overall population (high-risk: N = 5984; low/intermediate-risk: N = 10 471), the mean (median) duration of the observation period was 46.3 (43.8) months among the high-risk cohort and 47.8 (45.9) months among the low/intermediate-risk cohort. The mean (median) number of days receiving EBRT was 37.7 (44.0) days and 36.0 (41.0) days for patients in the high-risk or low/intermediate-risk cohort, respectively.

### Metastasis-free Survival

A higher proportion of patients in the high-risk cohort had progressed to metastasis or death, relative to patients in the low/intermediate-risk cohort by both 36 months (MFS rate: high-risk, 90.7%; low/intermediate-risk, 96.1%) and 60 months (high-risk, 82.6%; low/intermediate-risk, 91.7%) post-index. Compared with patients with low/intermediate-risk, patients with high-risk were 2.47 times more likely to develop metastasis or die by 36 months (HR [95% CI]: 2.47 [2.15, 2.84]; *P* < .001) and 2.29 times more likely by 60 months (HR [95% CI]: 2.29 [2.05, 2.55]; *P* < .001) (**Figure 2**). Results remained robust when analyzed among patients with ≥2 available risk classification components (**Supplementary Figure S2A**).

**Figure 2.**
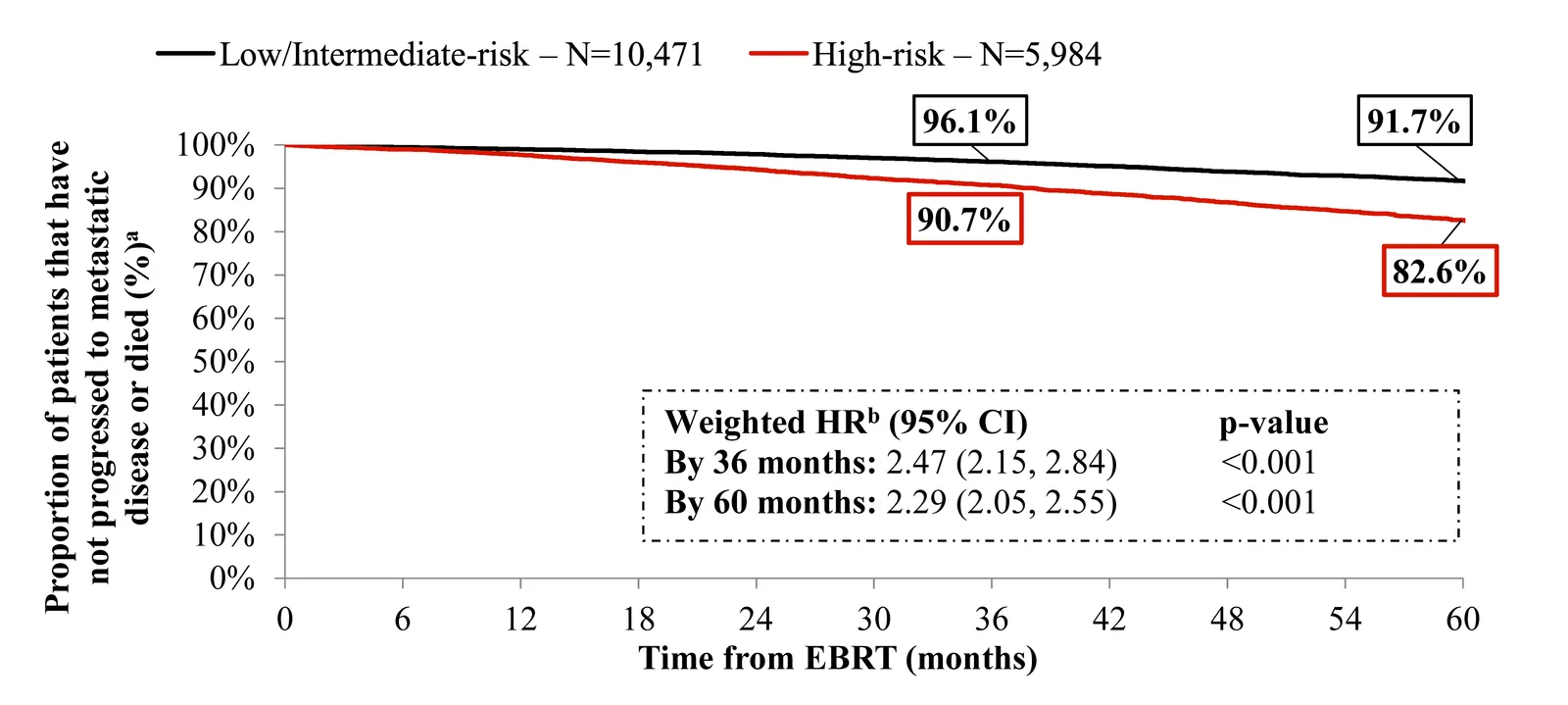
Metastasis-Free Survival Following Initiation of Treatment with EBRT in Patients with LPC Abbreviations: ARPI: androgen receptor pathway inhibitor; CI: confidence interval; EBRT: external beam radiation therapy; HR: hazard ratio; KRD+: Komodo Research Database; LPC: localized prostate cancer; PC: prostate cancer; PPS: Precision Point Specialty. ^a^Metastasis-free survival was assessed from the index date to the first date of metastasis or death. For patients still at risk, metastasis-free survival was censored at the latest of the open insurance claim activity in KRD+ or clinical activity in PPS, or end of data availability (ie, August 31, 2024). ^b^HRs and their associated 95% CIs and *P* values were estimated using weighted Cox proportional hazard models. Models were also adjusted with the following variables: age, race, geographic region, payer type, year of index date, time between PC diagnosis and index date, use of first-generation ARPI, use of bone anti-resorptive therapy, and Quan-Charlson Comorbidity Index score. Each patient was attributed an inverse-probability of treatment weight that was defined as follows: 1/(propensity score) for the high-risk cohort and 1/(1 − propensity score) for the low/intermediate-risk cohort. Normalized inverse probability of treatment weights were truncated at the 95th percentiles.

### Event-free Survival

Similarly, a higher proportion of patients in the high-risk cohort had progressed to metastasis, BCR, or death relative to patients in the low/intermediate-risk cohort by both 36 months (EFS rate: high-risk, 88.0%; low/intermediate-risk, 93.3%) and 60 months (high-risk, 78.4%; low/intermediate-risk, 88.1%) post-index. Compared with patients with low/intermediate-risk LPC, patients with high-risk LPC were 1.84 times more likely to have BCR, metastasis, or death by 36 months (HR [95% CI]: 1.84 [1.65, 2.06]; *P* < .001) and 1.90 times more likely at 60 months (HR [95% CI]: 1.90 [1.73, 2.08]; *P* < .001) post-index (**Supplementary Figure S3**). Results remained robust when analyzed among patients with ≥2 available risk classification components (**Supplementary Figure S2B**).

### Cost Outcomes

**Observation period:** within the cost outcomes population (high-risk: N = 980; low/intermediate-risk: N = 1862), the mean (median) observation period duration was 31.8 (28.1) months in the high-risk cohort and 32.7 (27.2) months in the low/intermediate-risk cohort.

Weighted mean [median] total all-cause healthcare costs were significantly higher in the high-risk cohort ($47 152 [$29 517] PPPY) vs the low/intermediate-risk cohort ($38 884 [$24 317] PPPY), representing a cost difference (95% CI) of $8269 (3869, 12 800) PPPY (*P* < .001; **Figure 3**). Patients in the high-risk vs low/intermediate-risk cohort incurred higher weighted mean all-cause pharmacy costs ($2274 vs $1941 PPPY; cost difference [95% CI]: $333 [−94 , 731] PPPY; *P* = .092) and significantly higher weighted mean [median] all-cause medical costs ($44 541 [$27 510] vs $36 690 [$22 355] PPPY; cost difference [95% CI]: $7850 [3558, 12 173] PPPY; *P* < .001). The observed difference in weighted mean total healthcare costs was primarily driven by significantly higher outpatient costs in the high-risk vs low/intermediate-risk cohort ($40 596 [$24 436] vs $33 054 [$19 707] PPPY; cost difference [95% CI]: $7543 [3409, 11 683]; *P* < .001).

**Figure 3.**
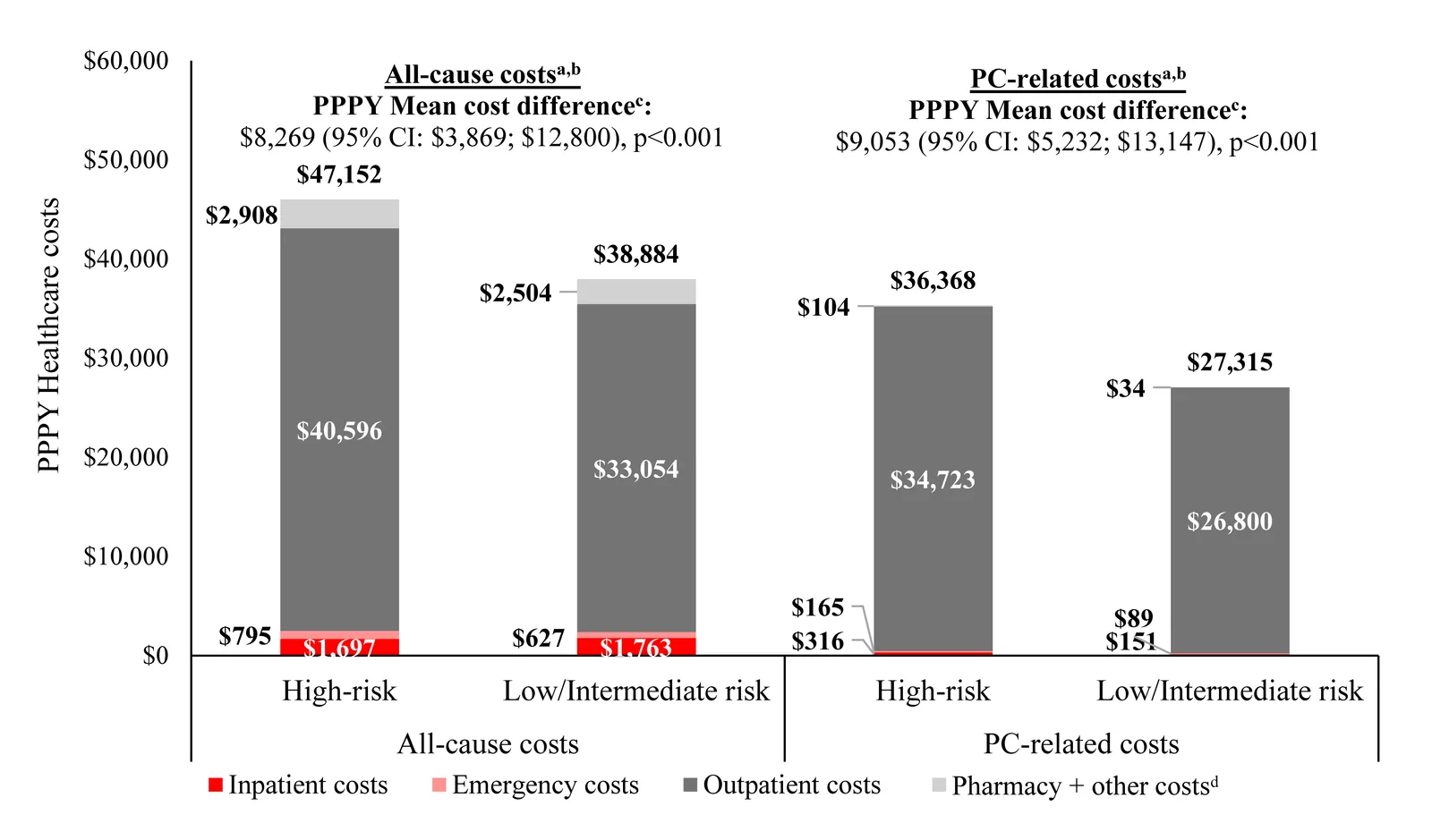
PPPY Costs Comparison During Complete Observation Period in Patients with LPC Treated with EBRT Abbreviations, CI, confidence interval; EBRT, external beam radiation therapy; LPC, localized prostate cancer; PC, prostate cancer; PPPY, per-patient-per-year; US, United States. ^a^Costs were inflated to 2024 US dollars. ^b^All costs were truncated at the 99th percentile to account for the influence of outliers. As a result, the sum of the individual mean cost differences may not align exactly with the total cost difference. ^c^95% CIs and *P* values for weighted mean cost differences were estimated using nonparametric bootstrapping procedures with 500 replications. ^d^Pharmacy costs and the costs of other services were reported together. Other services included medical claims for dental/vision care and durable medical equipment.

PC-related costs were significantly higher in the high-risk vs low/intermediate-risk cohort, including weighted mean [median] total PC-related healthcare costs ($36 368 [$19 456] vs $27 315 [$14 303] PPPY; cost difference [95% CI]: $9053 (5232, 13 147) PPPY; *P* < .001), weighted mean [median] PC-related pharmacy costs ($89 [$0] vs $26 [$0] PPPY; cost difference [95% CI]: $62 [24, 106] PPPY; *P* < .001), and weighted mean [median] PC-related medical costs ($35 718 [$18 815] vs $27 233 [$14 276] PPPY; cost difference [95% CI]: $8486 [4569, 12 381] PPPY; *P* < .001).

### LPC Period

The mean (median) duration of the LPC period was 30.0 (25.2) months in the high-risk cohort and 31.7 (26.0) months in the low/intermediate-risk cohort.

Weighted mean (median) total all-cause healthcare costs were $46 793 ($29 504) PPPY for patients in the high-risk cohort and $39 210 ($24 810) PPPY for patients in the low/intermediate-risk cohort (**Supplementary Figure S4**). Weighted mean (median) all-cause pharmacy costs were $2018 ($221) PPPY and $1877 ($229) PPPY in the high-risk and low/intermediate-risk cohorts, respectively, while weighted mean (median) all-cause medical costs were $44 691 ($27 374) PPPY and $37 034 ($22 733) PPPY, respectively.

Patients in the high-risk and low/intermediate-risk cohorts incurred $36 251 ($19 301) PPPY and $27 727 ($14 661) PPPY in weighted mean (median) total PC-related healthcare costs, with weighted mean (median) PC-related medical costs of $36 031 ($18 958) PPPY and $27 644 ($14 618) PPPY, respectively, accounting for >99% of total PC-related healthcare costs during the LPC period.

### Index EBRT Period

Weighted mean (median) total all-cause healthcare costs were $38 648 ($33 039) PPPY and $34 989 ($29 772) PPPY in the high-risk and low/intermediate-risk cohorts, respectively, with weighted mean (median) all-cause medical costs of $38 185 ($32 643) PPPY and $34 568 ($29 108) PPPY, respectively, accounting for >99% of total all-cause healthcare costs. Weighted mean (median) total PC-related healthcare costs were $36 821 ($31 335) PPPY in the high-risk cohort and $33 171 ($28 118) PPPY in the low/intermediate-risk cohort.

## DISCUSSION

Using linked EMR and administrative claims data, this real-world study evaluated clinical outcomes and healthcare costs among patients with high-risk vs low/intermediate-risk LPC treated with EBRT in real-world US clinical practice. Compared with patients with low/intermediate-risk LPC, those with high-risk LPC experienced a significantly higher risk of disease progression, metastasis, BCR, or death by both 36 and 60 months following EBRT initiation. Additionally, all-cause and PC-related healthcare costs were significantly higher in the high-risk cohort compared with the low/intermediate-risk cohort following EBRT initiation. Together, these findings reflect the inherently poorer prognosis and greater economic burden associated with high-risk LPC and suggest an unmet need for more effective treatment strategies in this patient population.

To our knowledge, only one study has previously evaluated real-world clinical outcomes following EBRT among patients with LPC, stratified by risk group.^10^ In that study, Surveillance, Epidemiology, and End Results (SEER)−Medicare data were analyzed for patients aged ≥65 years with newly diagnosed LPC or locally advanced PC who received EBRT as initial therapy.^10^ Patients were classified as either low/intermediate-risk (including very low-, low-, and intermediate-risk groups) or high-risk (including high- and very high-risk groups). Over a mean follow-up of more than 3.5 years, patients with high-risk LPC had nearly twice the risk of metastasis or death compared with those with low/intermediate-risk LPC (HR [95% CI]: 1.97 [1.78, 2.17]; *P* < .0001). These findings are consistent with those of the present study, whereby patients with high-risk vs low/intermediate-risk LPC had an approximately 2.5 times greater risk of metastasis or death at 3 years following EBRT initiation (HR [95% CI]: 2.47 [2.15, 2.84]; *P* < .001). In a separate real-world analysis of EMR data from the Veterans Health Administration, clinical outcomes were reported for more than 40 000 patients with LPC treated with definitive RT.^11^ Patients were stratified into low-, intermediate-, and high-risk groups, and 10-year outcomes, including overall survival, PC-specific survival, and MFS, worsened progressively with increasing risk status (all *P* < .001). Together, these findings highlight the link between high-risk status at LPC diagnosis and poorer clinical outcomes following EBRT in LPC. The current study underscores the value of using urology EMR data linked with administrative claims to provide a more complete patient profile for LPC risk classification and outcome assessment.

The present study provides novel insight into the healthcare costs associated with LPC treatment, revealing considerable all-cause and PC-related costs following EBRT. Specifically, patients with high-risk LPC incurred $8269 PPPY higher total all-cause healthcare costs compared with those with low/intermediate-risk LPC, driven primarily by higher outpatient costs in the high-risk cohort (*P* < .001). Direct medical costs associated with LPC management, stratified by patient risk status, have previously been estimated from the US commercial payer perspective using a decision-analytic model.^12^ In patients with LPC treated with definitive RT, cumulative per-patient costs were descriptively higher among those with high-risk vs low- or intermediate-risk LPC at both 5 years (low-risk: $51 914; intermediate-risk: $63 806; high-risk: $107 017) and 10 years (low-risk: $75 647; intermediate-risk: $130 466; high-risk: $213 882) following RT initiation. Though this study provides evidence linking higher healthcare costs with greater risk status in patients with LPC, future research among those treated with EBRT is warranted to corroborate and expand on the current findings.

Despite the considerable clinical and economic burden demonstrated among patients with high-risk LPC, progression to metastatic disease is associated with an even greater impact on both clinical outcomes and healthcare costs. High-risk patients with LPC are more likely to progress to metastatic disease, which is associated with significantly higher medical costs and healthcare resource use over 12 months compared with patients who do not progress.^16,17^ A prior study using linked SEER-Medicare data estimated that progression from localized stage to metastatic PC was associated with more than a $50 000 increase in annual healthcare costs during the first 12 months following metastasis.^18^ In another study that used administrative claims data, Trinh et al identified progression from localized to metastatic PC to be associated with a 3-fold increase in total direct all-cause healthcare costs over a mean follow-up period of 15 months.^19^ Furthermore, upon progression to metastatic PC, the 5-year survival rate was estimated to be 37.9%.^4^ Therefore, early monitoring in combination with more effective treatments is needed to reduce the burden among patients with LPC while preventing progression to metastatic PC.

The treatment landscape for LPC continues to evolve, with recent and ongoing clinical trials evaluating the effectiveness of both novel and existing agents for LPC management.^20–23^ Future real-world studies should aim to assess both the clinical and economic burden associated with these treatments in US clinical practice.

### Limitations

This study is associated with several limitations inherent to the use of real-world data. First, this study relied on EMR and administrative claims data, which may contain inaccuracies or omissions. The PPS component of the linked dataset did not include diagnoses, medical services, or prescriptions filled outside the PPS urology practice network. Although linkage with KRD+ allowed for the capture of relevant care received outside of the PPS network, including radiation oncology, imaging, and hospitalizations, some services may still not have been captured. Second, residual confounding following IPTW may have been present because of potential confounders not measured or not available in the data. Further, selection bias cannot be ruled out, as the choice to pursue EBRT rather than an alternative treatment may reflect unmeasured factors, such as patient preference or physician judgment. Third, given open-source insurance claims and EMR data were used in this study, patients’ complete medical histories, including diagnoses, procedures, or medication records, may not be complete as continuous insurance eligibility was not required for the overall study population. However, given most of LPC-related care is received in the urology practice setting, the key procedures, treatments, and outcomes would be captured. Fourth, the follow-up duration in this study is relatively short compared with the natural history of LPC. As such, the present results may understate both clinical event rates and cumulative cost differences between cohorts. Future studies with long-term follow-up are warranted to fully characterize outcomes and costs in this population. Fifth, risk classification relied on partial data when TNM stage, PSA level, and Gleason score were not all recorded within the 180 days pre-index. It should also be noted that observed differences reflect associations between LPC risk status and outcomes among patients treated with EBRT in routine clinical practice rather than causal effects of EBRT.

## CONCLUSION

In this real-world study, patients with high-risk LPC demonstrated significantly worse MFS and EFS following EBRT compared with those with low/intermediate-risk LPC, with a higher risk of disease progression, recurrence, or death. Additionally, patients with high-risk vs low/intermediate-risk LPC incurred greater healthcare costs following EBRT, driven primarily by outpatient costs. These findings reflect the substantial clinical and economic burden associated with high-risk LPC and underscore an unmet need for more effective treatment options among this patient population.

### Disclosures

N.S. is an employee of the Carolina Urologic Research Center and has received consulting fees from Johnson & Johnson, Bayer, Dendreon, Tolmar, Ferring, Medivation/Astellas, Amgen, Pfizer, AstraZeneca, Astellas Pharma, AbbVie, Merck, Bristol Myers Squibb/Sanofi, Exact Imaging, FerGene, InVitae, MDxHealth, Myriad Genetics, Propella Therapeutics, Genzyme, Sanofi, CG Oncology, Genesis Cancer Care, Urogen Pharma, Speciality Networks, PeerView, Clarity Pharmaceuticals, Lantheus Medical Imaging, Lilly, Photocure, Telix Pharmaceuticals, AIkido Pharma, Arquer Diagnostics, Asieris Pharmaceuticals, Minomic, Novartis, PlatformQ Health, Promaxo, Protara Therapeutics, Fize Medical, Accord Research, Antev, Aura Biosciences, Bioprotect, and Sumitomo Pharma Oncology. N.S. has received research funding from AbbVie, Amgen, Astellas Pharma, AstraZeneca, Bayer, Bristol Myers Squibb/Pfizer, Boston Scientific, Clovis Oncology, Dendreon, Exact Imaging, Ferring, Foundation Medicine, InVitae, Johnson & Johnson, MDxHealth, Merck, Myovant Sciences, Myriad Genetics, Nymox, Pfizer, Sanofi, Sesen Bio, Tolmar, CG Oncology, DisperSol, FORMA Therapeutics, Guardant Health, Jiangsu Yahong Meditech, Novartis, Pacific Edge, POINT Biopharma, Propella Therapeutics, SeaGen, MT Group, Theralase, Veru, Zenflow, Advantagene, Aragon Pharmaceuticals, Endocyte, Exelixis, FKD Therapies, Genentech, Istari Oncology, Medivation, OncoCellMDx, ORIC Pharmaceuticals, Palette Life Sciences, Plexxikon, RhoVac, Steba Biotech, Urogen Pharma, Urotronic, US Biotest, and Vaxiion for work performed outside of the current study. B.L. is an employee of Chesapeake Urology and has received consulting fees from Johnson & Johnson, Dendreon, Astellas Pharma, Bayer, AstraZeneca/MedImmune, Merck, Tolmar, AbbVie, Pfizer, and Myovant Sciences. L.K. has received consultant and speaker fees from Johnson & Johnson, Ferring, Astellas, Bayer, AstraZeneca and Pfizer and has participated in clinical trials with Johnson & Johnson, Bayer, Astellas, Ferring, Pfizer, Protara, and ImmunisAI. G.B. is an employee of New Jersey Urology and has received consulting fees from Johnson & Johnson. C.P. and S.B. are employees and stockholders of Johnson & Johnson. F.K., C.R., and D.P. are employees of Analysis Group, Inc., a consulting company that has provided paid consulting services to Johnson & Johnson, which funded the development and conduct of this study and manuscript. F.L. was an employee of Analysis Group, Inc., at the time the study was conducted.

### Human Ethics and Consent to Participate

The study was considered exempt research under 45 CFR § 46.104(d)(4) as it involved only the secondary use of data that were de-identified in compliance with the Health Insurance Portability and Accountability Act (HIPAA), specifically, 45 CFR § 164.514.

### Previous Presentations

Part of the material in this manuscript was presented as a poster at the Society of Urologic Oncology Annual Meeting, held December 2-5, 2025, in Phoenix, Arizona, USA.

## Supporting information

Online Supplementary Material

## Data Availability

The data that support the findings of this study are available from Precision Point Specialty Analytics (https://specialtynetworks.com/member-solution-pps-analytics/) and Komodo Health (https://www.komodohealth.com/). Restrictions apply to the availability of these data, which were used under license for this study.

## References

[1] American Cancer Society Key statistics for prostate cancer.

[2] Siegel R. L., Kratzer T. B., Giaquinto A. N., Sung H., Jemal A. (2025). Cancer statistics, 2025. CA Cancer J Clin.

[3] Wei J. T., Barocas D., Carlsson S.. (2023). Early detection of prostate cancer: AUA/SUO guideline part I: Prostate cancer screening. J Urol.

[4] National Cancer Institute, Surveillance, Epidemiology, and End Results Program Cancer Stat Facts: Prostate Cancer.

[5] (2025). Referenced with permission from the NCCN Clinical Practice Guidelines in Oncology (NCCN Guidelines®) for Prostate Cancer V.1.2025. © National Comprehensive Cancer Network, Inc. 2025. All rights reserved. Accessed September 17, 2025. To view the most recent and complete version of the guideline, go online to NCCN.org. NCCN makes no warranties of any kind whatsoever regarding their content, use or application and disclaims any responsibility for their application or use in any way.

[6] Almeeri M. N. E., Awies M., Constantinou C. (2024). Prostate cancer, pathophysiology and recent developments in management: a narrative review. Curr Oncol Rep.

[7] Eastham J. A., Auffenberg G. B., Barocas D. A.. (2022). Clinically localized prostate cancer: AUA/ASTRO guideline, part I: introduction, risk assessment, staging, and risk-based management. J Urol.

[8] Numakura K., Kobayashi M., Muto Y.. (2023). The current trend of radiation therapy for patients with localized prostate cancer. Curr Oncol.

[9] Achard V., Panje C. M., Engeler D., Zilli T., Putora P. M. (2021). Localized and locally advanced prostate cancer: treatment options. Oncology.

[10] Karsh L., Du S., He J., Waters D., Muser E., Shore N. (2024). Differences in real-world outcomes by risk classification for localized prostate cancer patients after radiation therapy. Prostate.

[11] Herr D. J., Elliott D. A., Duchesne G.. (2023). Outcomes after definitive radiation therapy for localized prostate cancer in a national health care delivery system. Cancer.

[12] Gustavsen G., Gullet L., Cole D., Lewine N., Bishoff J.T. (2020). Economic burden of illness associated with localized prostate cancer in the United States. Future Oncol.

[13] Shore N. D., Moul J. W., Pienta K. J., Czernin J., King M. T., Freedland S. J. (2024). Biochemical recurrence in patients with prostate cancer after primary definitive therapy: treatment based on risk stratification. Prostate Cancer Prostatic Dis.

[14] Dorfman A. H., Valliant R. (1997). The Hajek Estimator Revisited. American Statistical Association on Survey Research Methods.

[15] Austin P. C. (2009). Using the standardized difference to compare the prevalence of a binary variable between two groups in observational research. Commun Stat Simul Comput.

[16] Li T. T., Shore N. D., Mehra M.. (2017). Impact of subsequent metastases on costs and medical resource use for prostate cancer patients initially diagnosed with localized disease. Cancer.

[17] Pignot G., Maillet D., Gross E.. (2018). Systemic treatments for high-risk localized prostate cancer. Nature Rev Urol.

[18] Reddy S. R., Broder M. S., Chang E., Paydar C., Chung K. C., Kansal A. R. (2022). Cost of cancer management by stage at diagnosis among Medicare beneficiaries. Cur Med Res Opin.

[19] Trinh Q.-D., Chaves L. P., Feng Q., Zhu J., Sandin R., Abbott T. (2022). The cost impact of disease progression to metastatic castration-sensitive prostate cancer. J Manag Care Spec Pharm.

[20] Shore N., Hafron J., Saltzstein D.. (2024). Apalutamide for high-risk localized prostate cancer following radical prostatectomy (Apa-RP). J Urol.

[21] ClinicalTrials.gov A study of apalutamide in participants with high-risk, localized or locally advanced prostate cancer who are candidates for radical prostatectomy (PROTEUS).

[22] Zhang X., Zhou F., Lu T.. (2025). Neoadjuvant darolutamide plus androgen deprivation therapy for high-risk and locally advanced prostate cancer: a multicenter, open-label, single-arm, phase II trial. World J Urol.

[23] Turco F., Buttigliero C., Delcuratolo M. D.. (2024). Hormonal agents in localized and advanced prostate cancer: current use and future perspectives. Clin Genitourin Cancer.

