# Supplementary material for: Real-World Clinical and Economic Outcomes Among Patients with Localized Prostate Cancer Treated with External Beam Radiation Therapy Across Different Risk Stratifications in the United States
Source: J Health Econ Outcomes Res. 2026 Oct 30;13(2):222–30. doi: 10.36469/001c.171930 (PMC13724129; doi:10.36469/001c.171930)
Supplement: Supplementary file 1 [file jheor_2026_13_2_171930_373876.pdf]

Journal of Health Economics and Outcomes Research

Online Supplementary Material

Real-World Clinical and Economic Outcomes Among Patients with Localized Prostate Cancer Treated with External Beam Radiation Therapy Across Different Risk Stratifications in the United States.
JHEOR. 2026;13(2):222-230. doi:10.36469/jheor.2026.171930

Table S1: Risk Classification of Patients with LPC 1

Table S2: Risk Stratification Assessment 2

Table S3: Unweighted and Weighted Baseline Characteristics of Patients with LPC Treated with EBRT 3

Table S4: PPPY Weighted Costs in Baseline and Observation Periods in Patients with LPC Treated with EBRT 5

Figure S1: Study Design Scheme 7

Figure S2: Kaplan-Meier Rates for (A) Metastasis-Free Survival and (B) Event-Free Survival Among Patients With ≥2 Risk Components 8

Figure S3: Event-Free Survival Following Initiation of Treatment with EBRT in Patients with LPC 9

Figure S4: PPPY Weighted Costs During LPC Period in Patients with LPC Treated with EBRT 10

This supplementary material has been provided by the authors to give readers additional information about their work.

This is an open-access article distributed under the terms of the Creative Commons Attribution 4.0 International License (CCBY-4.0). View this license's legal deed at http://creativecommons.org/licenses/by/4.0 and legal code at http://creativecommons.org/licenses/by/4.0/legalcode for more information.

Table S1. Risk Classification of Patients with LPC

TNM Staging	Gleason Score	PSA	Disease risk stratification
T1a to T2a	2 to 6	<10 ng/mL	Low-risk
		10 to 20 ng/mL	Intermediate-risk
		>20 ng/mL	High-risk
	7	≤ 20 ng/mL	Intermediate-risk
		> 20 ng/mL	High-risk
	8 to 10	All PSA	High-risk
T2b and T2c	2 to 7	≤ 20 ng/mL	Intermediate-risk
		> 20 ng/mL	High-risk
	8 to 10	All PSA	High-risk
T3a and T3b	Any Gleason	All PSA	High-risk
T4	Any Gleason	All PSA	High-risk

Abbreviations: LPC, localized prostate cancer; PSA, prostate-specific antigen; TNM, Tumor, Node, Metastasis.

Table S2. Risk Stratification Assessment

	Overall	High-risk	Low/intermediate-risk			
			Low/intermediate-risk	Low-risk	Intermediate-risk	Low/intermediate-risk, unclassifiable
	**N=16,455**	**N=5,984**	**N=10,471**	**N=191**	**N=1,288**	**N=8,992**
**Patients with exactly 1 available risk stratification measure[a], n (%)**	2,056 (12.5)	2,056 (34.4)	0 (0.0)	0 (0.0)	0 (0.0)	0 (0.0)
TNM staging	31 (1.5)	31 (1.5)	0 (0.0)	0 (0.0)	0 (0.0)	0 (0.0)
PSA testing	155 (7.5)	155 (7.5)	0 (0.0)	0 (0.0)	0 (0.0)	0 (0.0)
Gleason score	1,870 (91.0)	1,870 (91.0)	0 (0.0)	0 (0.0)	0 (0.0)	0 (0.0)
**Patients with exactly 2 available risk stratification measures[a], n (%)**	12,348 (75.0)	3,386 (56.6)	8,962 (85.6)	0 (0.0)	0 (0.0)	8,962 (99.7)
TNM staging and PSA testing	54 (0.4)	54 (1.6)	0 (0.0)	0 (0.0)	0 (0.0)	0 (0.0)
TNM staging and Gleason score	320 (2.6)	320 (9.5)	0 (0.0)	0 (0.0)	0 (0.0)	0 (0.0)
PSA testing and Gleason score	11,974 (97.0)	3,012 (89.0)	8,962 (100.0)	0 (0.0)	0 (0.0)	8,962 (100.0)
**Patients with exactly 3 available risk stratification measures[a], n (%)**	2,051 (12.5)	542 (9.1)	1,509 (14.4)	191 (100.0)	1,288 (100.0)	30 (0.3)
**Patients with TNM staging information[b], n (%)**	2,456 (14.9)	947 (15.8)	1,509 (14.4)	191 (100.0)	1,288 (100.0)	30 (0.3)
T1	1,702 (10.3)	477 (8.0)	1,225 (11.7)	171 (89.5)	1,054 (81.8)	0 (0.0)
T2	581 (3.5)	297 (5.0)	284 (2.7)	20 (10.5)	234 (18.2)	30 (0.3)
T3	160 (1.0)	160 (2.7)	0 (0.0)	0 (0.0)	0 (0.0)	0 (0.0)
T4	13 (0.1)	13 (0.2)	0 (0.0)	0 (0.0)	0 (0.0)	0 (0.0)
**Patients with available PSA tests[b], n (%)**	14,234 (86.5)	3,763 (62.9)	10,471 (100.0)	191 (100.0)	1,288 (100.0)	8,992 (100.0)
Number of PSA tests, mean ± SD [median]	1.7 ± 1.1 [2.0]	1.4 ± 1.2 [1.0]	1.9 ± 1.0 [2.0]	1.8 ± 0.8 [2.0]	1.9 ± 1.0 [2.0]	1.9 ± 1.0 [2.0]
Most recent PSA level, ng/mL, n (%)						
<2 ng/mL, n (%)	1,955 (11.9)	721 (12.0)	1,234 (11.8)	17 (8.9)	83 (6.4)	1,134 (12.6)
≥2 to <4 ng/mL, n (%)	1,377 (8.4)	343 (5.7)	1,034 (9.9)	21 (11.0)	91 (7.1)	922 (10.3)
≥4 to <10 ng/mL, n (%)	7,550 (45.9)	1,293 (21.6)	6,257 (59.8)	153 (80.1)	854 (66.3)	5,250 (58.4)
≥10 to ≤20 ng/mL, n (%)	2,648 (16.1)	702 (11.7)	1,946 (18.6)	0 (0.0)	260 (20.2)	1,686 (18.8)
>20 ng/mL, n (%)	704 (4.3)	704 (11.8)	0 (0.0)	0 (0.0)	0 (0.0)	0 (0.0)
**Patients with available Gleason score[b], n (%)**	16,215 (98.5)	5,744 (96.0)	10,471 (100.0)	191 (100.0)	1,288 (100.0)	8,992 (100.0)
≤6	2,740 (16.7)	84 (1.4)	2,656 (25.4)	191 (100.0)	61 (4.7)	2,404 (26.7)
7	8,085 (49.1)	270 (4.5)	7,815 (74.6)	0 (0.0)	1,227 (95.3)	6,588 (73.3)
8	2,912 (17.7)	2,912 (48.7)	0 (0.0)	0 (0.0)	0 (0.0)	0 (0.0)
9	2,371 (14.4)	2,371 (39.6)	0 (0.0)	0 (0.0)	0 (0.0)	0 (0.0)
10	107 (0.7)	107 (1.8)	0 (0.0)	0 (0.0)	0 (0.0)	0 (0.0)

Abbreviations: PSA, prostate-specific antigen; SD, standard deviation; TNM, tumor node metastasis.
a. Risk was evaluated over the 6 months prior to initiation of definitive therapy.
b. Evaluated during the baseline period, up to and including the index date.

Table S3. Unweighted and Weighted Baseline Characteristics of Patients with LPC Treated with EBRT

	**Overall**						**Subset of patients with closed claims eligibility**					
	**Unweighted**			**Weighted[a,b]**			**Unweighted**			**Weighted[a,b]**		
	**High-risk**	**Low/ intermediate-risk**	**Std. Diff.[c]**	**High-risk**	**Low/ intermediate-risk**	**Std. Diff.[c]**	**High-risk**	**Low/ intermediate- risk**	**Std. Diff.[c]**	**High-risk**	**Low/ intermediate-risk**	**Std. Diff.[c]**
	**N=5,984**	**N=10,471**	**%**	**N=5,984**	**N=10,471**	**%**	**N=980**	**N=1,862**	**%**	**N = 980**	**N = 1,862**	**%**
**Age, mean ± SD [median]**	72.3 ± 7.3 [73.0]	69.7 ± 7.1 [70.0]	36.5*	70.8 ± 7.3 [71.0]	70.5 ± 7.2 [71.0]	4.4	70.7 ± 8.0 [71.0]	67.7 ± 7.5 [68.0]	**39.2***	68.9 ± 8.0 [69.0]	68.6 ± 7.6 [69.0]	5.0
**Race/ethnicity, n (%)**												
White	3,025 (50.6)	5,179 (49.5)	2.2	2,962 (49.5)	5,205 (49.7)	0.4	444 (45.3)	862 (46.3)	2.0	442 (45.1)	852 (45.8)	1.3
Black or African American	1,054 (17.6)	1,714 (16.4)	3.3	1,021 (17.1)	1,761 (16.8)	0.7	193 (19.7)	324 (17.4)	5.9	188 (19.2)	339 (18.2)	2.5
Asian or Pacific Islander	130 (2.2)	126 (1.2)	7.5	98 (1.6)	154 (1.5)	1.4	18 (1.8)	23 (1.2)	4.9	14 (1.4)	25 (1.4)	0.5
Hispanic or Latino	58 (1.0)	84 (0.8)	1.8	47 (0.8)	85 (0.8)	0.3	14 (1.4)	18 (1.0)	4.3	9 (1.0)	19 (1.0)	0.6
Unknown	1,717 (28.7)	3,368 (32.2)	7.6	1,856 (31.0)	3,267 (31.2)	0.4	311 (31.7)	635 (34.1)	5.0	326 (33.3)	626 (33.6)	0.7
**Geographic region, n (%)**												
South	3,219 (53.8)	5,712 (54.6)	1.5	3,255 (54.4)	5,688 (54.3)	0.1	455 (46.4)	880 (47.3)	1.7	466 (47.6)	880 (47.2)	0.7
Midwest	1,212 (20.3)	2,358 (22.5)	5.5	1,292 (21.6)	2,297 (21.9)	0.9	232 (23.7)	469 (25.2)	3.5	240 (24.5)	464 (24.9)	0.9
Northeast	1,041 (17.4)	1,725 (16.5)	2.5	1,000 (16.7)	1,746 (16.7)	0.1	215 (21.9)	359 (19.3)	6.6	194 (19.8)	367 (19.7)	0.3
West	511 (8.5)	673 (6.4)	8.0	436 (7.3)	737 (7.0)	1.0	78 (8.0)	153 (8.2)	1.0	79 (8.1)	150 (8.1)	0.0
**Payer type, n (%)**												
Medicare	4,825 (80.6)	7,731 (73.8)	16.3*	4,592 (76.7)	7,957 (76.0)	1.7	668 (68.2)	1,066 (57.3)	22.7*	605 (61.8)	1,126 (60.5)	2.7
Commercial	862 (14.4)	2,201 (21.0)	17.4*	1,083 (18.1)	1,979 (18.9)	2.1	245 (25.0)	652 (35.0)	22.0*	299 (30.5)	595 (31.9)	3.2
Medicaid	111 (1.9)	179 (1.7)	1.1	110 (1.8)	186 (1.8)	0.4	34 (3.5)	79 (4.2)	4.0	39 (4.0)	75 (4.0)	0.2
Unknown	186 (3.1)	360 (3.4)	1.9	200 (3.3)	349 (3.3)	0.1	33 (3.4)	65 (3.5)	0.7	37 (3.8)	66 (3.6)	1.1
**Year of EBRT procedure (index year), n (%)**												
2017	744 (12.4)	1,363 (13.0)	1.8	763 (12.7)	1,341 (12.8)	0.2	97 (9.9)	185 (9.9)	0.1	97 (9.9)	184 (9.9)	0.2
2018	818 (13.7)	1,541 (14.7)	3.0	840 (14.0)	1,501 (14.3)	0.9	151 (15.4)	287 (15.4)	0.0	145 (14.8)	285 (15.3)	1.3
2019	960 (16.0)	1,523 (14.5)	4.2	907 (15.2)	1,568 (15.0)	0.5	157 (16.0)	262 (14.1)	5.5	148 (15.1)	273 (14.6)	1.2
2020	829 (13.9)	1,401 (13.4)	1.4	818 (13.7)	1,417 (13.5)	0.4	143 (14.6)	251 (13.5)	3.2	140 (14.3)	257 (13.8)	1.5
2021	1,028 (17.2)	1,710 (16.3)	2.3	1,007 (16.8)	1,739 (16.6)	0.6	160 (16.3)	315 (16.9)	1.6	164 (16.7)	312 (16.7)	0.0
2022	903 (15.1)	1,575 (15.0)	0.1	913 (15.2)	1,584 (15.1)	0.4	166 (16.9)	323 (17.3)	1.1	174 (17.8)	326 (17.5)	0.8
2023	688 (11.5)	1,315 (12.6)	3.3	720 (12.0)	1,284 (12.3)	0.7	104 (10.6)	236 (12.7)	6.4	109 (11.1)	224 (12.1)	3.1
2024	12 (0.2)	38 (0.4)	3.1	16 (0.3)	33 (0.3)	0.9	2 (0.2)	3 (0.2)	1.0	2 (0.2)	2 (0.1)	2.6

	Overall						Subset of patients with closed claims eligibility					
	Unweighted			Weighted[a,b]			Unweighted			Weighted[a,b]		
	High-risk	Low/ intermediate-risk	Std. Diff.[c]	High-risk	Low/ intermediate-risk	Std. Diff.[c]	High-risk	Low/ intermediate- risk	Std. Diff.[c]	High-risk	Low/ intermediate-risk	Std. Diff.[c]
	N=5,984	N=10,471	%	N=5,984	N=10,471	%	N=980	N=1,862	%	N = 980	N = 1,862	%
**Time between initial PC diagnosis and index date, months, mean ± SD [median]**	9.8 ± 20.0 [3.7]	13.5 ± 25.4 [3.6]	16.4*	11.6 ± 23.2 [3.9]	12.4 ± 23.5 [3.6]	3.5	9.9 ± 18.8 [3.8]	13.6 ± 26.3 [3.6]	16.3*	11.5 ± 21.4 [3.9]	12.6 ± 24.4 [3.6]	4.6
**Prior use of ADT, n (%)**	4,915 (82.1)	4,447 (42.5)	89.7*	4,859 (81.2)	4,668 (44.6)	81.9*	799 (81.5)	746 (40.1)	93.8*	791 (80.8)	786 (42.2)	86.3*
**Use of first-generation ARPI, n (%)**	900 (15.0)	571 (5.5)	32.0*	549 (9.2)	823 (7.9)	4.7	188 (19.2)	112 (6.0)	40.5*	109 (11.1)	172 (9.2)	6.3
**Use of bone anti-resorptive therapy, n (%)**	340 (5.7)	175 (1.7)	21.4*	194 (3.2)	264 (2.5)	4.3	38 (3.9)	24 (1.3)	16.4*	23 (2.4)	36 (1.9)	3.0
**Quan-Charlson Comorbidity Index, mean ± SD [median]**	3.4 ± 1.8 [3.0]	3.2 ± 1.7 [3.0]	13.3*	3.3 ± 1.7 [3.0]	3.2 ± 1.7 [3.0]	1.8	3.7 ± 2.0 [3.0]	3.5 ± 1.8 [3.0]	13.5*	3.6 ± 1.9 [3.0]	3.5 ± 1.9 [3.0]	2.4

Abbreviations: ADT, androgen deprivation therapy; ARPI, androgen receptor pathway inhibitor; EBRT, external beam radiation therapy; LPC, localized prostate cancer; PC, prostate cancer; SD, standard deviation; Std Diff, standardized difference.

a. The propensity score was obtained from a logistic regression model where index treatment was the dependent variable and with the following baseline characteristics as independent variables: age, race, geographic region, payer type, year of index date, time between PC diagnosis and index date, use of first-generation ARPI, use of bone anti-resorptive therapy, and Quan-Charlson comorbidity index score.
b. The number of patients reported in this weighted population represents the sum of weights for the corresponding non-weighted patients, rounded to the nearest integer. The proportions displayed were calculated before the rounding and may be slightly different than if they were calculated based on rounded numbers.
c. A Std. Diff. of <10% was considered well balanced.
* Std. Diff. ≥10%.

Table S4. PPPY Weighted Costs in Baseline and Observation Periods in Patients with LPC Treated with EBRT[a]

	Weighted costs[a-d]							
	Baseline period[e]		Observation period[f]		LPC period[g]		Total index EBRT episode costs (first 90 days)[h]	
	High-risk	Low/intermediate-risk	High-risk	Low/intermediate-risk	High-risk	Low/intermediate-risk	High-risk	Low/intermediate-risk
**Length of period, months, mean ± SD [median]**	12.0 ± 0.0 [12.0]	12.0 ± 0.0 [12.0]	31.8 ± 22.4 [28.1]	32.7 ± 22.4 [27.2]	30.0 ± 21.9 [25.2]	31.7 ± 21.9 [26.0]	2.9 ± 0.4 [3.0]	2.9 ± 0.3 [3.0]
**All-cause and PC-related costs[i], PPPY, mean ± SD [median]**								
**All-cause medical and pharmacy costs**	14,557 ± 11,927 [11,416]	13,017 ± 11,264 [10,030]	47,152 ± 51,846 [29,517]	38,884 ± 44,963 [24,317]	46,793 ± 51,585 [29,504]	39,210 ± 44,978 [24,810]	38,648 ± 25,328 [33,039]	34,989 ± 22,514 [29,772]
**All-cause pharmacy costs**	693 ± 1,723 [41]	757 ± 1,910 [41]	2,274 ± 5,282 [235]	1,941 ± 4,536 [231]	2,018 ± 4,496 [221]	1,877 ± 4,185 [229]	444 ± 1,198 [11]	389 ± 1,101 [11]
**All-cause medical costs**	13,801 ± 11,528 [10,819]	12,100 ± 10,627 [9,319]	44,541 ± 50,941 [27,510]	36,690 ± 44,031 [22,355]	44,691 ± 51,210 [27,374]	37,034 ± 44,051 [22,733]	38,185 ± 25,278 [32,643]	34,568 ± 22,449 [29,108]
Inpatient costs	618 ± 3,407 [0]	439 ± 2,935 [0]	1,697 ± 6,190 [0]	1,763 ± 6,245 [0]	1,546 ± 5,951 [0]	1,750 ± 6,241 [0]	221 ± 1,577 [0]	169 ± 1,443 [0]
Emergency room costs	291 ± 1,015 [0]	271 ± 946 [0]	795 ± 1,776 [0]	627 ± 1,659 [0]	779 ± 1,784 [0]	628 ± 1,689 [0]	252 ± 990 [0]	178 ± 874 [0]
Outpatient costs	12,232 ± 9,064 [10,067]	10,665 ± 8,384 [8,587]	40,596 ± 48,633 [24,436]	33,054 ± 41,272 [19,707]	40,921 ± 49,040 [24,358]	33,408 ± 41,330 [20,135]	37,120 ± 24,415 [31,668]	33,716 ± 21,771 [28,731]
Other services[j] costs	397 ± 655 [206]	417 ± 672 [235]	634 ± 1,556 [32]	563 ± 1,294 [21]	628 ± 1,607 [27]	566 ± 1,333 [20]	80 ± 265 [0]	68 ± 212 [0]
**PC-related medical and pharmacy costs**	8,579 ± 6,699 [6,818]	7,268 ± 6,226 [5,666]	36,368 ± 47,654 [19,456]	27,315 ± 39,140 [14,303]	36,251 ± 47,538 [19,301]	27,727 ± 39,202 [14,661]	36,821 ± 24,219 [31,335]	33,171 ± 21,761 [28,118]
**PC-related pharmacy costs**	1 ± 6 [0]	1 ± 5 [0]	89 ± 547 [0]	26 ± 291 [0]	26 ± 192 [0]	13 ± 134 [0]	0 ± 3 [0]	0 ± 2 [0]
**PC-related medical costs**	8,539 ± 6,665 [6,759]	7,240 ± 6,186 [5,663]	35,718 ± 47,084 [18,815]	27,233 ± 39,116 [14,276]	36,031 ± 47,518 [18,958]	27,644 ± 39,177 [14,618]	36,754 ± 24,218 [31,127]	33,141 ± 21,736 [28,117]
Inpatient costs	106 ± 820 [0]	62 ± 631 [0]	316 ± 1,616 [0]	151 ± 1,096 [0]	232 ± 1,335 [0]	145 ± 1,045 [0]	14 ± 102 [0]	5 ± 63 [0]
Emergency room costs	31 ± 206 [0]	29 ± 196 [0]	165 ± 671 [0]	89 ± 514 [0]	156 ± 653 [0]	88 ± 511 [0]	131 ± 579 [0]	74 ± 443 [0]
Outpatient costs	8,040 ± 6,212 [6,420]	6,801 ± 5,724 [5,363]	34,723 ± 46,587 [18,266]	26,800 ± 38,939 [13,972]	35,129 ± 47,065 [18,354]	27,204 ± 39,007 [14,246]	36,080 ± 23,837 [30,601]	32,783 ± 21,408 [27,959]
Other services[j] costs	212 ± 274 [104]	203 ± 258 [119]	15 ± 72 [0]	8 ± 55 [0]	12 ± 63 [0]	8 ± 51 [0]	7 ± 45 [0]	4 ± 31 [0]

Abbreviations: ARPI, androgen receptor pathway inhibitor; BCR, biochemical recurrence; EBRT, external beam radiation therapy; ICD-10-CM, International Classification of Diseases, 10th Revision, Clinical Modifications; KRD+, Komodo Research Database; LHRH, luteinizing hormone-releasing hormone; LPC, localized prostate cancer; PC, prostate cancer; PPPY, per-patient-per-year; SD, standard deviation; US, United States.

a. Costs are inflated to 2024 US dollars using the medical care component of the consumer price index and were reported PPPY.

b. Costs are reported for the duration of each disease stage until progression to the next disease stage among the closed claims population.

c. Propensity scores were generated using probability estimates from a logistic regression models using the following predictors: age, race, geographic region, payer type, year of index date, time between PC diagnosis and index date, use of first-generation ARPI, use of bone anti-resorptive therapy, and baseline Quan-Charlson comorbidity index. Each patient was attributed an inverse-probability of treatment weight that was defined as follows: 1/(propensity score) for the high-risk cohort and 1/(1-propensity score) for the low/intermediate-risk cohort. Normalized inverse-probability of treatment weights were truncated at the 95th percentiles.

d. All cost variables were truncated at the 99th percentile to account for the influence of extreme outliers with unusually high costs.

e. The baseline period was defined as the 12-months prior to the index date.

f. The observation period was defined as the time between the index date and the end of continuous insurance eligibility in KRD+.

g. The overall LPC period was defined as the portion of the observation period that occurred prior to evidence of either metastasis, castration-resistance or BCR. If none of these events were observed, the LPC period was defined as time from index date until the end of the observation period.

h. The total index EBRT episode costs were reported as total costs incurred during the first 90 days of follow-up including the index date.

i. PC-related costs were identified with the ICD-10-CM code C61, or a claim with a procedure code for LHRH, LPC therapy, or other advanced therapies for PC.

j. Other services include medical claims for dental/vision care and durable medical equipment.

Figure S1. Study Design Scheme

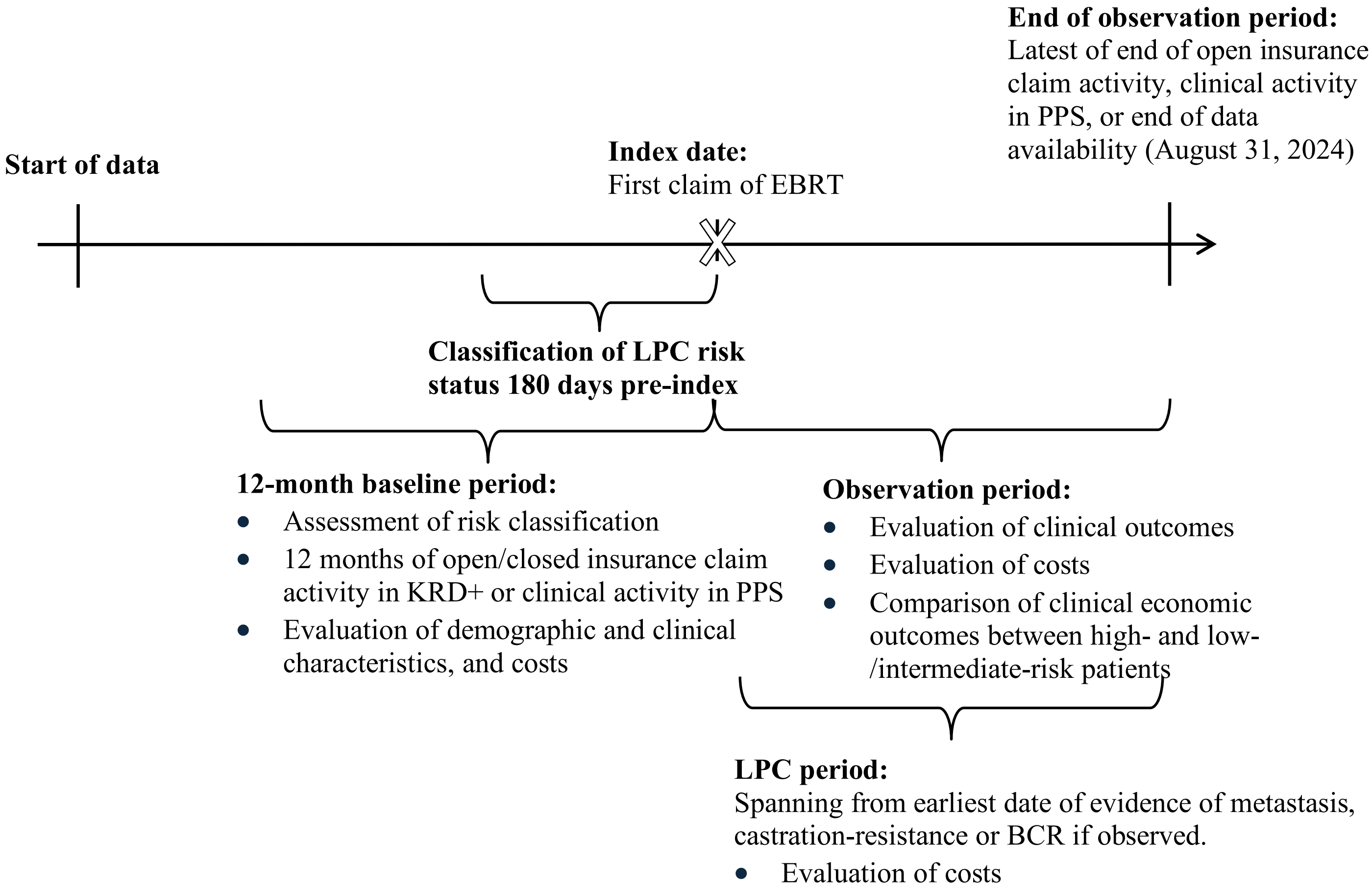

Abbreviations: BCR, biochemical recurrence; EBRT, external beam radiation therapy; KRD+, Komodo Research Database; LPC, localized prostate cancer; PPS, Precision Point Specialty.

Figure S2. Kaplan-Meier Rates for (A) Metastasis-Free Survival and (B) Event-Free Survival Among Patients with ≥2 Risk Components[a,b]

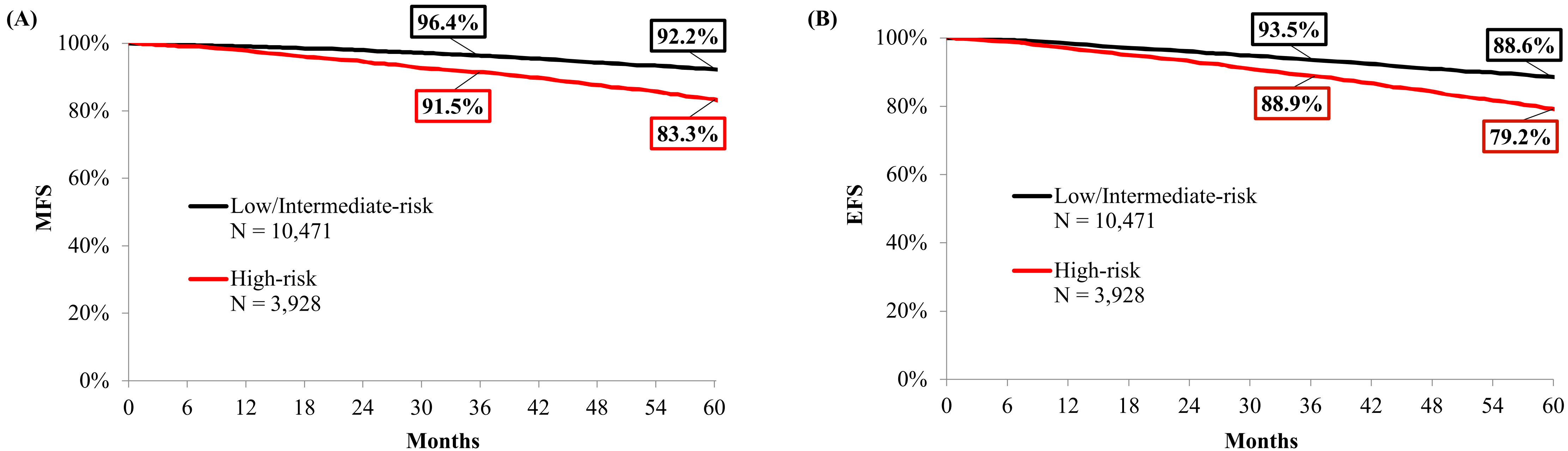

Abbreviations: BCR, biochemical recurrence; EFS, event-free survival; MFS, metastasis-free survival; KRD+, Komodo Research Database; PPS, Precision Point Specialty.

a. Metastasis-free survival was assessed from the index date to the earliest date of metastasis or death. For patients still at risk, metastasis-free survival was censored at the latest of end of open insurance claim activity in KRD+ or clinical activity in PPS, or end of data availability (i.e., 31 August 2024).

b. Event-free survival was assessed from the index date to the earliest date of BCR, metastasis or death. For patients still at risk, event-free survival was censored at the latest of end of open insurance claim activity in KRD+ or clinical activity in PPS, or end of data availability (i.e., 31 August 2024).

Figure S3. Event-Free Survival Following Initiation of Treatment with EBRT in Patients with LPC

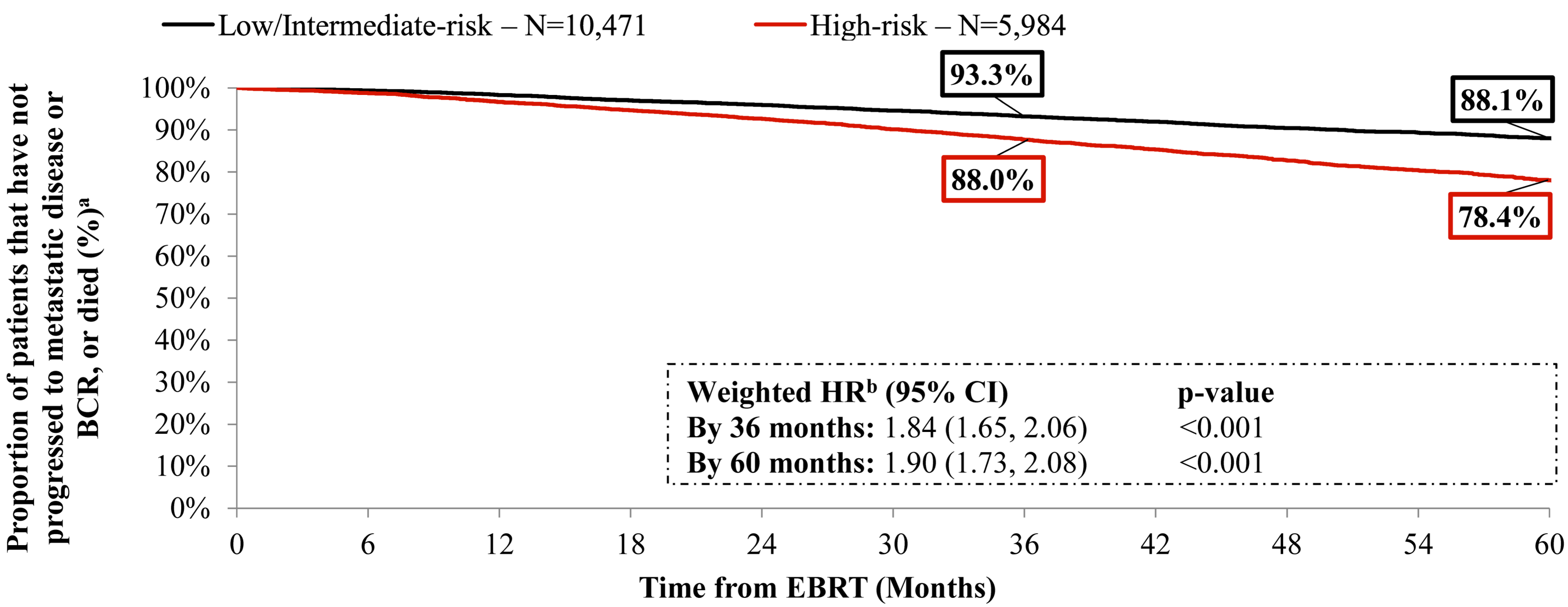

Abbreviations: ARPI, androgen receptor pathway inhibitor; BCR, biochemical recurrence; CI, confidence interval; EBRT, external beam radiation therapy; HR, hazard ratio; KRD+, Komodo Research Database; LPC, localized prostate cancer; PC, prostate cancer; PPS, Precision Point Specialty.

a. Event-free survival was assessed from the index date to the earliest date of BCR, metastasis or death. For patients still at risk, event-free survival was censored at the latest of end of open insurance claim activity in KRD+ or clinical activity in PPS (including death), or end of data availability (i.e., 31 August, 2024).

b. HRs and their associated 95% CIs and p-values were estimated using weighted Cox proportional hazard models. Models were also adjusted with the following variables: age, race, geographic region, payer type, year of index date, time between PC diagnosis and index date, use of first-generation ARPI, use of bone anti-resorptive therapy, and Quan-Charlson comorbidity index score. Each patient was attributed an inverse-probability of treatment weight that was defined as follows: 1/(propensity score) for the high-risk cohort and 1/(1-propensity score) for the low/intermediate-risk cohort. Normalized inverse-probability of treatment weights were truncated at the 95th percentiles.

Figure S4. PPPY Weighted Costs During LPC Period in Patients with LPC Treated with EBRT[a,b,c]

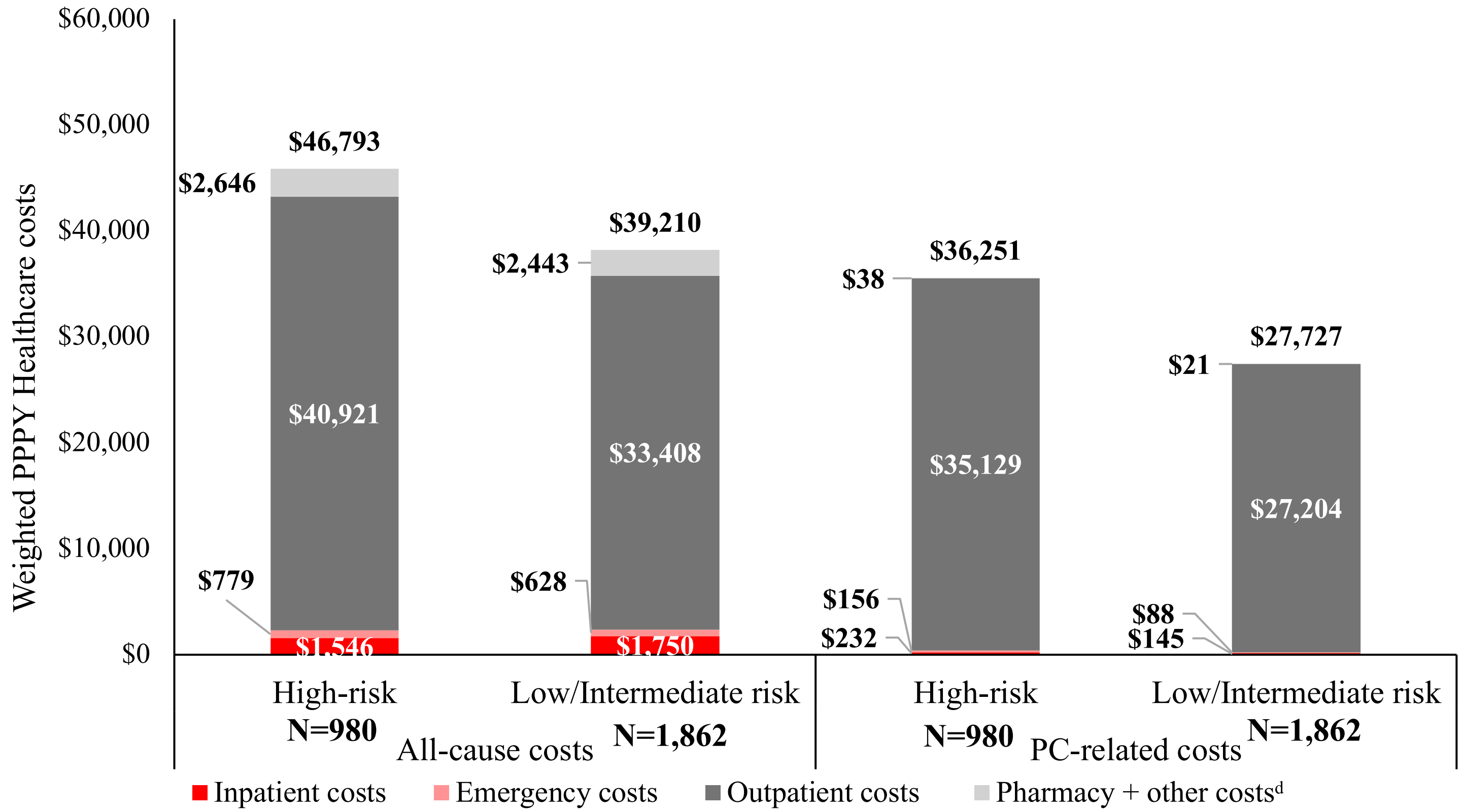

Abbreviations: BCR, biochemical recurrence; EBRT, external beam radiation therapy; LPC, localized prostate cancer; PC, prostate cancer; PPPY, per-patient-per-year; US, United States.

a. Costs were inflated to 2024 US dollars.

b. All costs were truncated at the 99th percentile to account for the influence of outliers.

c. The overall LPC period was defined as the portion of the observation period that occurred prior to evidence of either metastasis, castration-resistance, or BCR. If none of these events were observed, the LPC period was defined as time from index date until the end of the observation period.

d. Pharmacy costs and the costs of other services were reported together. Other services included medical claims for dental/vision care and durable medical equipment.
